# Brazilian *Copaifera* Species: Antifungal Activity against Clinically Relevant *Candida* Species, Cellular Target, and In Vivo Toxicity

**DOI:** 10.3390/jof6030153

**Published:** 2020-08-28

**Authors:** Géssica Andrade, Haniel Chadwick Silva Orlando, Liliana Scorzoni, Reginaldo Santos Pedroso, Fariza Abrão, Marco Túlio Menezes Carvalho, Rodrigo Cassio Sola Veneziani, Sérgio Ricardo Ambrósio, Jairo Kenupp Bastos, Maria José Soares Mendes-Giannini, Carlos Henrique Gomes Martins, Regina Helena Pires

**Affiliations:** 1 University of Franca, Franca 14404-600, Brazil; gessicaandrade16123@gmail.com (G.A.); hanny_chad@hotmail.com (H.C.S.O.); rpedroso@ufu.br (R.S.P.); farizaabrao@yahoo.com.br (F.A.); marcotulioicb@outlook.com (M.T.M.C.); rodrigo.veneziani@unifran.edu.br (R.C.S.V.); sergio.ambrosio@unifran.edu.br (S.R.A.); 2 School of Pharmaceutical Sciences, São Paulo State University (UNESP), Araraquara 14801-902, Brazil; liliana.scorzoni@ict.unesp.br (L.S.); giannini@fcfar.unesp.br (M.J.S.M.-G.); 3 Science and Technology Institute of São José dos Campos (ICT), São Paulo State University (UNESP), São José dos Campos 12245-000, Brazil; 4 Health Technical School (ESTES), Federal University of Uberlandia, Uberlandia 38400-732, Brazil; 5 Faculty of Pharmaceutical Sciences of Ribeirao Preto, University of Sao Paulo, Ribeirão Preto 14040-903, Brazil; jkbastos@fcfrp.usp.br; 6 Institute of Biomedical Sciences (ICBIM), Federal University of Uberlandia, Uberlandia 38400-902, Brazil

**Keywords:** *Copaifera*, *Candida*, antifungal, *C. elegans*–*Candida* infected model

## Abstract

Plants belonging to the genus *Copaifera* are widely used in Brazil due to their antimicrobial properties, among others. The re-emergence of classic fungal diseases as a consequence of antifungal resistance to available drugs has stimulated the search for plant-based compounds with antifungal activity, especially against *Candida*. The *Candida*-infected *Caenorhabditis elegans* model was used to evaluate the in vitro antifungal potential of *Copaifera* leaf extracts and trunk oleoresins against *Candida* species. The *Copaifera* leaf extracts exhibited good antifungal activity against all *Candida* species, with MIC values ranging from 5.86 to 93.75 µg/mL. Both the *Copaifera paupera* and *Copaifera reticulata* leaf extracts at 46.87 µg/mL inhibited *Candida glabrata* biofilm formation and showed no toxicity to *C. elegans.* The survival of *C. glabrata*-infected nematodes increased at all the tested extract concentrations. Exposure to *Copaifera* leaf extracts markedly increased *C. glabrata* cell vacuolization and cell membrane damage. Therefore, *Copaifera* leaf extracts are potential candidates for the development of new and safe antifungal agents.

## 1. Introduction

Although modern medicine is well developed in most parts of the world, WHO recognizes that a large part of the population in developing countries depends on traditional medicine for primary care [1]. In this regard, Brazil is the most biodiverse country on the planet and holds valuable traditional knowledge associated with the use of medicinal plants [2].

*Copaifera* (*Leguminosae* Juss., subfamily *Caesalpinoideae* Kunth) is a genus of large trees that comprises more than 72 species occurring in Latin America, India, and West Africa. Sixteen of these species are found only in Brazil, mainly in the northern and northeastern regions, in the states of Amazonas, Pará, and Ceará [3,4]. Plant derivatives such as oleoresins and essential oils are widely employed in folk medicine; the use of leaf extracts is less common, but some reports have demonstrated their antiurolithiac, antiedematogenic, and gastroprotective potential, which were mostly related to the presence of galloylquinic acid derivatives and flavonoids [5,6,7].

In the last three decades, antifungal resistance has caused great concern and stimulated studies for the discovery of new antifungal agents, especially against *Candida* spp., given that classical fungal diseases have re-emerged due to the development of antifungal resistance. *Candida albicans* is the most prevalent species among *Candida* infections, but other species such as *C. glabrata, C. parapsilosis, C. tropicalis*, and *C. krusei* (*Pichia kudriavzevii/Issatchenkia orientalis*) also underlie human infections [8,9]. Even though *C. parapsilosis* currently consists of a complex of three related species, namely, *C. parapsilosis* sensu lato, *C. orthopsilosis*, and *C. metapsilosis*, Brazilian isolates of the latter species have been reported at a lower frequency [10]. In addition, some *Candida* species, like *C. glabrata* and *C. krusei*, have different degrees of susceptibility and may be resistant to antifungals that are employed in clinical practice [11].

However, the biocompatibility of new products or products of unknown action should be analyzed by in vitro cell culture studies and animal experiments before such products are applied to humans. Studies on invertebrates have been conducted more frequently, and the use of the nematode *Caenorhabditis elegans* to investigate infections, virulence, toxicity, and antifungal drug activity [12] is noteworthy.

In view of the emergence of resistant strains and the expansion of fungal virulence mechanisms, we evaluate the *Candida* species susceptibility profile against *Copaifera* species extracts and oleoresins by determining their minimal inhibitory concentration values, their ability to inhibit biofilm formation, and their effect when associated with amphotericin B. In addition, the potential cellular target, as well as the toxicity of these plant-derived products, was assessed.

## 2. Materials and Methods

### 2.1. Plant Material

The *Copaifera* oleoresins and leaf extracts were obtained and analyzed for their chemical compositions, as previously described by our group [5]. Briefly, the *C. duckei, C. langsdorffii, C. paupera, C. reticulata, C. trapezifolia,* and *C. pubiflora* oleoresins were collected from holes made in the center of the tree trunks. The leaf extracts were prepared by macerating the ground air-dried *C. duckei*, *C. langsdorffii*, *C. lucens*, *C. multijuga*, *C. oblongifolia*, *C. paupera*, *C. reticulata*, and *C. trapezifolia* leaves in 7:3 ethanol/water at room temperature for 48 h. The extracts were concentrated under vacuum and lyophilized. Then, the plant-derived products were chemically characterized by UPLC–MS/MS (oleoresins) and HPLC (leaf extracts), as previously published by our group [5].

### 2.2. Microorganisms

The *Candida* strains were acquired from the American Type Culture Collection (ATCC) and included *C. albicans* ATCC 5314, *C. glabrata* ATCC 2001, *C. parapsilosis* ATCC 22019, *C. krusei* ATCC 6258, and *C. tropicalis* ATCC 13803. The strains were cryopreserved at −80 °C. At the time of the assays, they were inoculated in Sabouraud dextrose agar (SBA, Difco Laboratories, Detroit, MI, USA) and Chromagar *Candida* (Becton Dickinson and Company, Sparks, MD, USA) at 37 °C for 48 h in order to evaluate their purity and viability.

### 2.3. Antifungal Activity Evaluation

The methodology was based on the studies published by Santos et al. [13] and the Clinical Laboratory Standards Institute (CLSI) protocols M27-A2 S4 [14] and M60 [15]. The leaf extracts or oleoresins were solubilized in dimethyl sulfoxide (2% DMSO, *v*/*v*) and diluted in Roswell Park Memorial Institute (RPMI) 1640 medium. The tested concentrations ranged from 1.46 to 750 μg/mL. Yeast-containing cell suspensions were prepared and diluted in RPMI to reach a final concentration of 0.5 × 10^3^ to 2.5 × 10^3^ CFU/mL. Each yeast cell suspension was inoculated into 96-well microplates that had previously been prepared with the dilutions of the *Copaifera* samples, which was followed by incubation at 37 °C for 48 h. The wells containing only inoculum and culture medium were considered as 100% organism viability. The addition of 0.015% aqueous rezasurin solution and further incubation at 37 °C for 3 h helped to visualize yeast viability [16]. The minimum inhibitory concentration (MIC) was defined as the lowest concentration of antifungal agent that maintained blue staining in the supernatant medium. Controls consisting of 2% DMSO (*v*/*v*), RPMI medium without inoculum, and plant extracts diluted in RPMI were also included. The *C. krusei* ATCC 6258 and *C. parapsilosis* ATCC 22,019 strains and amphotericin B (AMB) at concentrations ranging from 0.015 to 8 μg/mL were employed as test quality controls [14]. Plant-derived products are considered good, moderate, weak, and inactive antimicrobials when they display MIC values lower than 100 µg/mL, from 100 to 500 µg/mL, from 500 to 1000 µg/mL, and above 1000 µg/mL, respectively [17,18]. The tests were performed in triplicate, and the mean value was used as a result. These tests allowed us to select the most active *Copaifera* species and the most susceptible *Candida* species.

### 2.4. Checkerboard Assay

In clinical practice, AMB has been the standard fungicidal in the treatment of severe fungal infections. Therefore, we evaluated the effect of AMB combined with the *Copaifera* leaf extracts by the checkerboard method [19]. The tested AMB final concentrations and *Copaifera* sample concentrations ranged from 0.03 to 16 µg/mL [14] and from 3.90 to 500 µg/mL, respectively. The assay was carried out with a final inoculum of 2 × 10^3^ CFU/mL [20], and the plates were incubated at 37 °C for 48 h. A 0.015% aqueous rezasurin solution was used to assess fungal cell viability. The Fractional Inhibitory Concentration Index (FICI), which is defined as the sum of MIC of each drug in combination divided by MIC of the drug alone, was calculated. Synergy, additivity, indifference, and antagonism were defined as FICI ≤ 0.5, 0.5 < FICI < 1, 1 ≤ FICI < 4, and FICI ≥ 4, respectively [19]. The tests were conducted in triplicate.

### 2.5. Copaifera Species Effect on the Inhibition of Candida Biofilm Formation

For these assays, only the most active *Copaifera* leaf extracts were selected based on the results obtained in the MIC and FICI assays. A suspension prepared by equitable mixing of the standardized *Candida* cell suspension (10^6^ CFU/mL) and *Copaifera* extract, which had previously been diluted in RPMI at concentrations ranging from 2.78 to 750 μg/mL, were inoculated into 96-well microplates. Then, the plates were statically incubated at 37 °C for 48 h. After incubation, the supernatant was aspirated, the wells were washed with PBS, and viable cells were determined by the tetrazolium salt (sodium 3′-[1-(phenylaminocarbonyl)-3,4-tetrazolium]-bis(4-methoxy-6-nitro) hydroxylated benzene sulfonic acid—XTT) reduction assay [21]. The effective *Copaifera* extract concentration that was able to inhibit biofilm formation was determined by the reduction of approximately 80% (MIBC_80_) of the optical density as compared to the control that was free of the chemical substance (100% of survivors).

### 2.6. Copaifera Species Effect against Preformed C. glabrata Biofilms

For these tests, only the most active extracts (*C. paupera* and *C. reticulata*) and the most sensitive *Candida* species (*C. glabrata*) were used. The biofilms were formed according to a previously published protocol [21]. After incubation at 37 °C for 24 h, the biofilms were washed with PBS (3×), and the *Copaifera* extract was added at concentrations ranging from 2.78 to 750 µg/mL, which was followed by incubation for additional 24 h at 37 °C. The biofilms were washed (PBS 3×), and the viability was measured by the XTT assay. The effective *Copaifera* leaf extract concentration that was able to reduce ≥80% OD, as compared to the control that was free of extract (100% of survivors), was considered as MBEC_80_. Each assayed experimental condition was performed in triplicate, and the arithmetic mean of the results was used.

### 2.7. Sample Preparation for Transmission Electron Microscopy

The cells were prepared for observation by transmission electron microscopy (TEM) in agreement with previously published protocol [22]. Briefly, the *C. glabrata* cells were grown in SDA agar for 24 h. Two cultivar loops were transferred to Falcon-type conical tubes containing 20 mL of YPD broth (yeast peptone dextrose: 1% yeast extract, 2% peptone, 2% dextrose) added with the *Copaifera* spp leaf extract at the determined 0.5 × MIC and 1 × MIC concentrations. The tubes were placed in an orbital shaker (150–180 rpm) at 30 °C for 18 to 24 h. The cells were centrifuged, and the pellet was fixed with 2.5% (*v*/*v*) glutaraldehyde in 0.1 M sodium cacodylate buffer (pH 7.4). Secondary fixation was carried out with 1% osmium tetroxide aqueous solution, and the cells were treated with 2% uranyl acetate for embedding. After dehydration with gradients of ethanol (50%, 75%, 95%, and 100%) and propylene oxide, the cells were included in Spurr resin before being subjected to ultrathin microtome cuts. The samples were contrasted with uranyl acetate and examined under an electron transmission microscope (Zeiss Libra 120, Oberkochen, Germany, acquisition voltage: 60 kv, emission: 10 µA). AMB at 2 µg/mL was employed as a control drug.

### 2.8. Experiments with the Animal Model Caenorhabditis Elegans

The *C. elegans* AU37 (*glp*-4 (*bn*2) I; *sek*-1 (*km*4) X) mutant strain, obtained from the *Caenorhabditis* Genetics Center (CGC, University of Minnesota), was used in all the experiments. The strain was grown on agar plates seeded with *Escherichia coli* OP50, which were incubated at 15 °C [12,22]. The tested *Candida* strain was grown in SDA broth (Difco) at 35 °C under shaking. A 100 μL aliquot of this culture was inoculated into a solid BHI medium (Difco) containing kanamycin (90 mg/mL) and ampicillin (200 mg/mL), which was followed by incubation at 30 °C for 24 h. Synchronized nematodes in stage L4 were inoculated on the agar plates containing grown yeast strains and incubated at 25 °C for 3 h [23].

In parallel, the L4 worms were plated on agar plates containing grown *E. coli* OP50. After incubation for 3 h, the worms were washed with M9 buffer (3.0 g KH_2_ PO_4_, 6.0 g Na_2_HPO_4_, 0.5 g NaCl, 1.0 g NH_4_Cl, and H_2_O to complete 1 L). Next, they were transferred to 12-well plates containing 1 mL of a solution consisting of 60% M9 buffer, 40% BHI broth (Difco), 10 mg/mL cholesterol in ethanol, 200 mg/mL ampicillin, and 90 mg/mL kanamycin. Approximately 10–12 nematodes were placed in each well. To evaluate the antifungal efficacy, selected *Copaifera* extracts at selected concentrations (0.5 MIC, 1 × MIC, and 2 × MIC) and AMB (1 mg/mL) were added to the solution. For the toxicity tests, the nematodes in stage L4 were placed in contact with different extract concentrations. Three independent experiments were conducted for each treatment. The survival curves were analyzed with the software Graph-Pad Prism 5 (La Jolla, CA, USA). The Kaplan–Meier method and the level of significance calculated by the log-rank test (Mantel–Cox) were used. A *p*-value higher than 0.05 was considered significant [24].

## 3. Results and Discussion

Targeted research into the discovery of new drugs from natural products has proven to be an alternative of growing interest for scientists and the pharmaceutical industry. Products obtained from the *Copaifera* tree, such as oleoresins, essential oils, and leaf extracts, have shown relevant results in experiments related to antibacterial activity, but studies related to its antifungal activity are scarce [13,25,26,27,28].

According to the adopted criterion, all the *Copaifera* leaf extracts were active against all the tested *Candida* species (Table 1). *Copaifera reticulata* and *C. paupera* were the most effective and provided a MIC value of 5.86 μg/mL against *C. glabrata*, the most sensitive *Candida* species (Table 1). *C. krusei* and *C. orthopsilosis* followed as the most sensitive *Candida* species (Table 1). For both species, MIC values of 11.72 μg/mL were achieved with the *Copaifera paupera* and *C. reticulata* extracts against *C. krusei* and with the *Copaifera duckei* and *C. lucens* extracts against *C. orthopsilosis*.

*Candida glabrata* and *C. krusei* have intrinsic resistance to fluconazole [29]. Therefore, the *Copaifera* species should be further investigated as an alternative treatment for these *Candida* infections.

**Figure 1:**
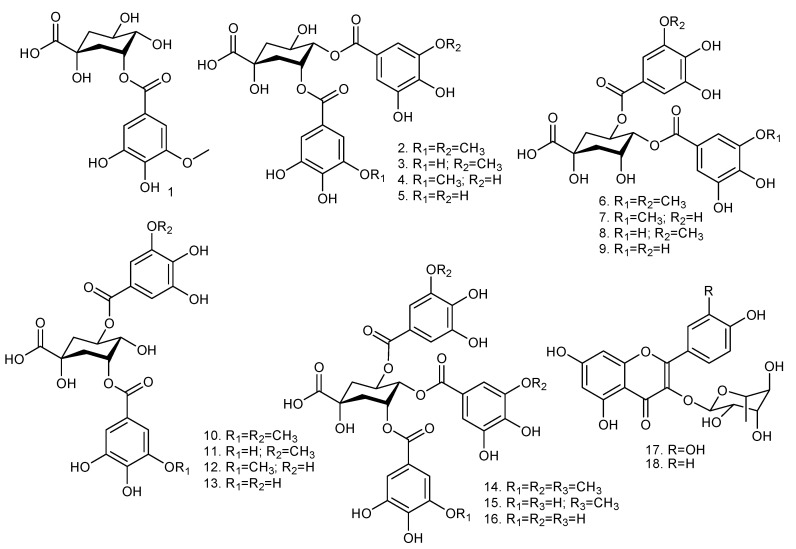
Chemical structures of the galloylquinic acids and flavonoids present in *Copaifera* species: 3-*O*-(3-*O*-methyl galloyl) quinic acid (**1**); 3,4-di-*O*-(3-*O*-methyl galloyl) quinic acid (**2**); 3-*O*-(galloyl)-4-*O*-(3-*O*-methyl galloyl) quinic acid (**3**); 3-*O*-(3-*O*-methyl galloyl)-4-*O*-(galloyl) quinic acid (**4**); 3,4-di-*O*-(galloyl) quinic acid (**5**); 4,5-di-*O*-(3-*O*-methyl galloyl) quinic acid (**6**); 4-*O*-(3-*O*-methyl galloyl)-5-*O*-(galloyl) quinic acid (**7**); 4-*O*-(galloyl)-5-*O*-(3-*O*-methyl galloyl) quinic acid (**8**); 4,5-di-*O*-(galloyl) quinic acid (**9**); 3,5-di-*O*-(3-*O*-methyl galloyl) quinic acid (**10**); 3-*O*-(galloyl)-5-*O*-(3-*O*-methyl galloyl) quinic acid (**11**); 3-*O*-(3-*O*-methyl galloyl)-5-*O*-(galloyl) quinic acid (**12**); 3,5-di-*O*-(galloyl) quinic acid (**13**); 3,4,5-tri-*O*-(3-*O*-methyl galloyl) quinic acid (**14**); 3,5-di-*O*-(galloyl)-4-*O*-(methyl galloyl) quinic acid (**15**); 3,4,5-tri-*O*-(galloyl) quinic acid (**16**); quercetrin (**17**); afzelin (**18**). Adapted from Furtado et al. [5].

Our research group has previously reported that the leaf extracts of *Copaifera* spp. are rich in galloylquinic acids and flavonoids (Figure 1) [5]. De Leo et al. [30] reported that phenolic compounds, mostly galloylquinic acid derivatives and flavonoids, were isolated from *Baseonerna acuminatum* P. Choux (*Asclepiadaceae*). According to these authors, 2-methoxy-5-(1′,2′,3′-trihydroxypropyl)-phenyl-1-*O*-(6″-galloyl)-beta-d-glucopyranoside, 2-methoxy-5-hydroxymethyl-phenyl-1-*O*-(6″-galloyl)-beta-d-glucopyranoside, benzyl 6′-*O*-galloyl-beta-d-glucopyranoside, and 1,6-di-*O*-galloyl-beta-d-glucopyranose were active against clinical and ATCC *Candida albicans* strains. Li et al. [31] reported that a flavonoid derivative known as mattucinol-7-*O*-[4″,6″-*O*-(S)-hexahydroxydiphenoyl]-beta-d-glucopyranoside inhibited the aspartic proteases (SAPs) secreted by *Candida albicans*, which are a major virulence factor in *Candida* infections. Another study showed that the *C. glabrata* species was the most sensitive to the *Camellia sinensis* leaf extract, which is rich in galloyl derivatives [32]. Other studies have also indicated the potential of polyphenolic-rich extracts [33,34,35] against pathogenic fungi, suggesting that the MIC values listed in Table 1 may be associated with the *Copaifera* leaf extract polyphenolic content.

As shown in Table 1, higher concentrations of the evaluated *Copaifera* oleoresins were required to inhibit *Candida* growth. Only the *C. paupera* oleoresin showed moderate antifungal activity against all the tested *Candida* species, and we achieved the best inhibition results with 187.5 μg/mL *C. paupera* oleoresin against *C. glabrata* and *C. krusei*. Santos et al. [13] reported similar moderate antifungal results for the *C. paupera* oleoresin, which displayed moderate activity against the dermatophyte fungi *Trichophyton rubrum* and *Microsporum canis*, with MIC values of 250 and 500 μg/mL, respectively.

By considering the best results obtained with the *Copaifera* leaf extracts, these derivatives were studied to understand other aspects of their antifungal activity. The Fractional Inhibitory Concentration Index (FICI) results revealed that the interaction between the investigated extracts and AMB was not promising because the FICI values ranged from 1.5 to 4 (Table 2). The extracts of some *Copaifera* species, like *C. duckei*, *C. oblongifolia*, and *C. trapezifolia*, presented antagonism when combined with AMB, that is, there was a decrease in AMB MIC when compared to the MIC value of AMB alone (Table 2). Combined with AMB, *Copaifera paupera* provided the lowest FICI (1.5) against all the tested *Candida* species (Table 2).

Microorganisms with a planktonic phenotype (that is, responsive to host defenses and therapeutics) can switch to a life form (biofilm) that is able to adapt in order to survive in an aggressive host tissue environment. This has assumed a specific meaning in the pathogenesis of infections and has been thoroughly studied by Costerton et al. [36]. Over the years, studies focusing on adhesion inhibition and biofilm formation have gained progressive importance [37].

In this context, it was investigated how the *Copaifera* extracts affected *Candida* biofilm formation inhibition. For most of the evaluated extracts, the MICB_80_ values did not reveal activity. The exceptions were the *C. paupera* and *C. reticulata* leaf extracts, which inhibited *Candida glabrata* biofilm formation at MICB_80_ = 46.87 µg/mL. Nevertheless, in the tested concentration range (2.78 to 750 µg/mL), the *C. paupera* and *C. reticulata* leaf extracts were not effective against preformed *C. glabrata* biofilms (MBEC_80_). Studies addressing *Copaifera* antifungal activity are scarce [13]. However, decreased biofilm sensitivity to antifungal compounds has been attributed to the properties of biofilm cells, including the expression of virulence genes, mRNA, and proteins and the production of an extracellular matrix that acts as a physical barrier to drug penetration and environmental adversities [38].

Infections caused by the opportunistic pathogen *C. glabrata* may be related to biofilm formation and resistance to commonly used antifungal agents [39,40]. The fact that both *C. paupera* and *C. reticulata* leaf extracts at 46.87 μg/mL inhibited *C. glabrata* biofilm formation (MBIC_80_) demonstrates an anti-*C. glabrata* effect with respect to surface adsorption. Attachment is the initial stage in biofilm formation. During this stage, most cells are still in the fluid phase and exhibit the same planktonic cell phenotype, so they are more susceptible to antimicrobial agents [41].

In addition, the position and quantity of surface cell wall mannoproteins and the presence of adhesin-like proteins contribute to the yeast hydrophobic state, allowing its initial attachment to solid surfaces or tissues and favoring its ability to form and maintain biofilms [42,43]. The *C. glabrata* cell walls, the only medically important species that does not form pseudohyphae, display more β-1,2-linked mannose residues [44] and adhesin-like cell wall proteins, which are incorporated in different stages of yeast growth [43]. It was inferred that the *Copaifera* extract may have interfered with the incorporation of these adhesins into the *C. glabrata* cell wall during biofilm development.

**Figure 2:**
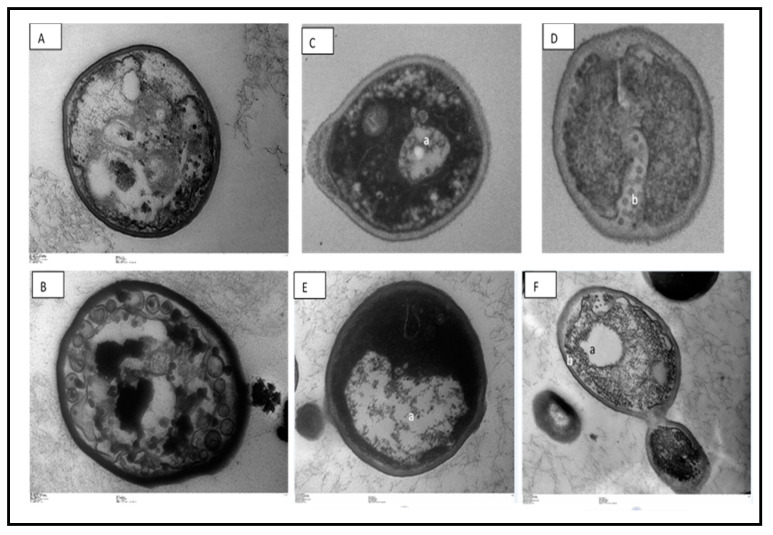
Cellular alterations in *Candida glabrata* cells after exposure to *Copaifera* leaf extracts, observed with transmission electron microscopy. (**A**)—control (untreated), (**B**)—treatment with 2 µg/mL amphotericin, (**B**,**C**)—0.5 × MIC *Copaifera paupera*, (**D**)—0.5 × MIC *Copaifera reticulata*, (**E**)—1 × MIC *C. paupera*, and (**F**)—1 × MIC *C. reticulata* leaf extracts. There is intense vacuolization (a) and plasma membrane destruction (b). Magnification (**A**,**B**,**D**): 25,000×; (**C**,**E**): 20,000×, (**F**): 5000×.

Transmission electron microscopy revealed a large increase in *C. glabrata* cell vacuolization, as well as cell membrane damage (Figure 2). Changes in conformation and rupture of the plasma membrane can contribute to cell death. The *Copaifera* plant has already been reported for its property of damaging the cell wall of bacterial pathogens, especially *Staphylococcus aureus* [13]. However, here, the yeast cell wall appeared intact, which could be explained by the difference in the structural components of the fungal wall in relation to the bacterial wall. Future tests will be carried out to elucidate cell permeability completely.

**Figure 3:**
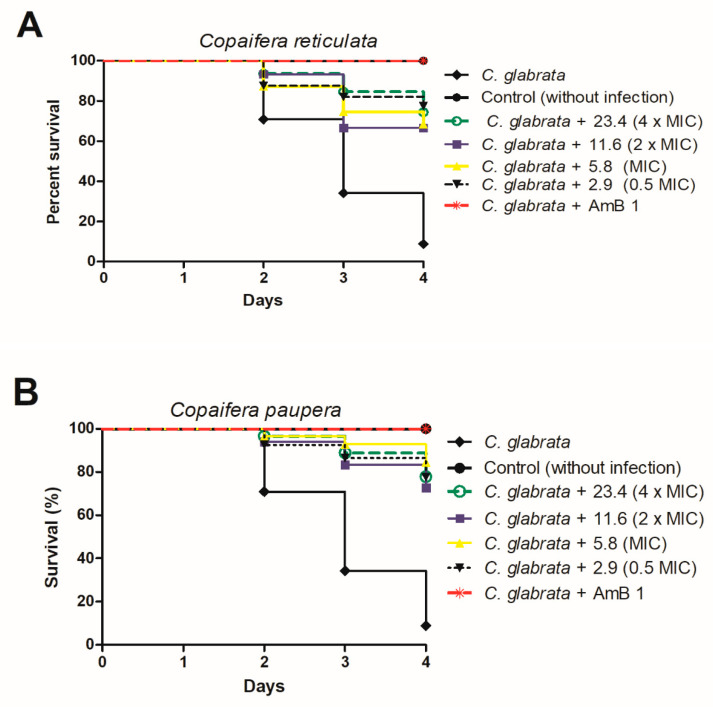
Survival curve of *C. elegans* infected with *C. glabrata* ATCC 2001 and treated with concentrations equivalent to 0.5 MIC, MIC, 2 × MIC, and 4 × MIC of *Copaifera reticulata* (**A**) and *Copaifera paupera* (**B**). Amphotericin B at 1 µg/mL was used as control. Statistical differences were observed between the groups treated with all the *C. reticulata* concentrations and the nontreated control group (*C. glabrata*) (*p* < 0.0001). In addition, statistical differences were observed between the groups treated with all the *C. paupera* concentrations and the nontreated control group (*C. glabrata*) (*p* < 0.0001). Log-rank test (Mantel–Cox).

The use of invertebrate hosts has recently arisen and facilitated studies on fungal pathogenesis. Among these hosts, the nematode *C. elegans* stands out and has been successfully used to study the toxicity and effectiveness of substances against some fungi [12,23,45]. Therefore, we evaluated the toxicity to *C. elegans* of *Copaifera reticulata* and *C. paupera* extracts at sub-MIC (0.5 × MIC) concentration, equivalent to 2.9 µg/mL, at MIC of 5.8 µg/mL, and supra-MIC (2 × MIC, 4 × MIC) concentrations, equivalent to 11.6 and 23.4 µg/mL, respectively (Figure 3).

All the *C. reticulata* extract concentrations (A) increased *C. elegans* survival by between 68% and 75% as compared to infected and untreated larvae (Figure 3). Similarly, treatment with *C. paupera* (B) provided survival percentages higher than 75% (Figure 3). Previously, we had reported the absence of genotoxic effects on the micronucleus frequencies of Chinese hamster lung fibroblast (V79) cell cultures and even a cytoprotective effect of *Copaifera* spp leaf extracts, which we associated with the large number of phenolic compounds in the extracts [5,46]. All these data indicated the efficacy and safe use of *Copaifera* leaf extracts against *C. glabrata* infection.

## 4. Conclusions

According to the results, namely, the lack of in vivo toxicity and the potent antifungal effect of *Copaifera paupera* and *C. reticulata* leaf extracts, these extracts should be further studied in order to develop new drugs, especially drugs designed for the treatment of *Candida glabrata* infections.

## Figures and Tables

**Table 1:** Minimal inhibitory concentrations of the hydroalcoholic leaf extracts and oleoresins obtained from *Copaifera* species against *Candida* species (MIC values in µg/mL).

*Copaifera* Leaf Extract	*C. albicans* (ATCC 5314)	*C. glabrata* (ATCC 2001)	*C. krusei* (ATCC 6258)	*C. parapsilosis* (ATCC 22019)	*C. orthopsilosis* (ATCC 96141)	*C. tropicalis* (ATCC 13803)
*C. duckei*	46.87	23.43	23.43	23.43	11.72	46.87
*C. langsdorffii*	93.75	23.43	23.43	93.73	46.87	93.75
*C. lucens*	46.87	46.87	46.87	46.87	11.72	46.87
*C. multijuga*	46.87	23.43	23.43	46.87	46.87	46.87
*C. oblongifolia*	93.75	23.43	23.43	46.87	23.43	93.75
*C. paupera*	46.87	5.86	11.72	23.43	23.43	46.87
*C. reticulata*	46.87	5.86	11.72	23.43	23.43	46.87
*C. trapeziofolia*	93.75	23.43	46.87	46.87	23.43	93.75
*Copaifera* Oleoresin						
*C. duckei*	>750.00	>750.00	>750.00	>750.00	>750.00	>750.00
*C. langsdorffii*	>750.00	750.00	750.00	750.00	750.00	>750.00
*C. paupera*	750.00	187.50	187.50	750.00	750.00	750.00
*C. pubiflora*	>750.00	750.00	750.00	>750.00	750.00	>750.00
*C. reticulata*	>750.00	750.00	375.00	750.00	750.00	>750.00
*C. trapeziofolia*	>750.00	375.00	750.00	750.00	375.00	>750.00
Amphotericin B			1.00	0.25		

**Table 2:** Interactions between *Copaifera* leaf extracts and amphotericin B against *Candida* spp. planktonic cells.

Strains	MIC in Combination (µg/mL)							
	Cd/AMB	Cla/AMB	Clu/AMB	Cm/AMB	Co/AMB	Cp/AMB	Cr/AMB	Ct/AMB
*C. albicans* ATCC 5314	93.75/1.0	93.75/0.5	46.87/1.0	46.87/1.0	187.5/2.0	46.87/0.25	46.87/0.5	187.5/1.0
*C. glabrata* ATCC 2001	23.43/1.0	11.72/0.5	46.87/1.0	11.72/0.7	46.87/1.0	5.86/0.25	5.86/0.5	23.43/1.0
*C. krusei* ATCC 6258	23.43/2.0	11.72/1.0	46.87/2.0	11.72/1.0	46.87/2.0	11.72/0.5	11.72/1.0	93.73/2.0
*C. parapsilosis* ATCC 22019	46.87/0.5	93.73/0.5	46.87/0.5	23.43/0.5	93.73/0.5	23.43/0.12	23.43/0.25	93.73/0.5
*C. orthopsilosis* ATCC 96141	23.43/0.5	46.87/0.5	11.72/0.5	23.43/0.5	93.73/0.5	23.43/0.125	23.43/0.25	46.87/0.5
*C. tropicalis* ATCC 13803	93.75/1.0	93.75/1.0	46.87/1.0	46.87/1.0	187.5/2.0	46.87/0.25	46.87/0.5	187.5/1.0
	**FICI (Interpretation)**							
	**Cd**	**Cla**	**Clu**	**Cm**	**Co**	**Cp**	**Cr**	**Ct**
*C. albicans* ATCC 5314	4.0/A	2.0/I	3.0/I	3.0/I	6.0/A	1.5/I	2.0/I	4.0/A
*C. glabrata* ATCC 2001	4.0/A	1.5/I	3.0/I	2.40/I	4.0/A	1.5/I	2.0/I	3.0/I
*C. krusei* ATCC 6258	4.0/A	1.5/I	3.0/I	1.5/I	4.0/A	1.5/I	2.0/I	4.0/I
*C. parapsilosis* ATCC 22019	4.0/A	3.0/I	3.0/I	2.49/I	3.9/I	1.5/I	2.0/I	4.0/A
*C. orthopsilosis* ATCC 96141	4.0/A	3.0/I	3.0/I	2.49/I	6.0/A	1.5/I	2.0/I	4.0/A
*C. tropicalis* ATCC 13803	4.0/A	3.0/I	3.0/I	3.0/I	6.0/A	1.5/I	2.0/I	4.0/A

Cd: *Copaifera duckei*, Cla: C. *landsdorffii,* Clu: *C. lucens*, Cm: *C. multijuga*, Co: *C. oblongifolia*, Cp: *C. paupera*, Cr: C. *reticulata*, Ct: *C. trapezifolia*, AMB: amphotericin B, I: indifferent, A: antagonism.
